# A learning robot for cognitive camera control in minimally invasive surgery

**DOI:** 10.1007/s00464-021-08509-8

**Published:** 2021-04-27

**Authors:** Martin Wagner, Andreas Bihlmaier, Hannes Götz Kenngott, Patrick Mietkowski, Paul Maria Scheikl, Sebastian Bodenstedt, Anja Schiepe-Tiska, Josephin Vetter, Felix Nickel, S. Speidel, H. Wörn, F. Mathis-Ullrich, B. P. Müller-Stich

**Affiliations:** 1 Department for General, Visceral and Transplantation Surgery, Heidelberg University Hospital, Im Neuenheimer Feld 420, 69120 Heidelberg, Germany; 2 Health Robotics and Automation Laboratory, Institute for Anthropomatics and Robotics, Karlsruhe Institute of Technology, Karlsruhe, Germany; 3 Department for Translational Surgical Oncology, National Center for Tumor Diseases, Partner-Site Dresden, Dresden, Germany; 4 Centre for International Student Assessment (ZIB) e.V., TUM School of Education, Technical University of Munich, Munich, Germany

**Keywords:** Cognitive surgical robotics, Artificial intelligence, Surgical data science, Colorectal surgery, Machine learning

## Abstract

**Background:**

We demonstrate the first self-learning, context-sensitive, autonomous camera-guiding robot applicable to minimally invasive surgery. The majority of surgical robots nowadays are telemanipulators without autonomous capabilities. Autonomous systems have been developed for laparoscopic camera guidance, however following simple rules and not adapting their behavior to specific tasks, procedures, or surgeons.

**Methods:**

The herein presented methodology allows different robot kinematics to perceive their environment, interpret it according to a knowledge base and perform context-aware actions. For training, twenty operations were conducted with human camera guidance by a single surgeon. Subsequently, we experimentally evaluated the cognitive robotic camera control. A VIKY EP system and a KUKA LWR 4 robot were trained on data from manual camera guidance after completion of the surgeon’s learning curve. Second, only data from VIKY EP were used to train the LWR and finally data from training with the LWR were used to re-train the LWR.

**Results:**

The duration of each operation decreased with the robot’s increasing experience from 1704 s ± 244 s to 1406 s ± 112 s, and 1197 s. Camera guidance quality (good/neutral/poor) improved from 38.6/53.4/7.9 to 49.4/46.3/4.1% and 56.2/41.0/2.8%.

**Conclusions:**

The cognitive camera robot improved its performance with experience, laying the foundation for a new generation of cognitive surgical robots that adapt to a surgeon’s needs.

**Supplementary Information:**

The online version contains supplementary material available at 10.1007/s00464-021-08509-8.

Medical robots have gained increasing popularity as assistive devices for surgical applications throughout the last decades [1], especially in laparoscopic surgery. In surgical specialties as diverse as urology [2], gynecology [3], and general surgery [4] the da Vinci® telemanipulator (Intuitive Surgical, Sunnyvale, USA) is leading the field. Whereas the da Vinci® combines several robotic arms in an overall system to perform a complete operation via robotic telemanipulation, a number of smaller, specialized systems have been proposed for specific applications, thus far mainly focusing on camera guidance in laparoscopic surgery [5–9]. Learning systems have been of particular interest to leverage data for learning complex skills [10, 11].

In laparoscopic surgery, camera guidance is of utmost importance for clinical outcome and successful avoidance of complications as the surgeon’s view of the operating field is determined by the quality of images and strongly influenced by camera motion. Nevertheless, in the standard of care, camera guidance in laparoscopic surgery is not performed by the operating surgeon, but by a surgical assistant. The assistant is required to understand and anticipate the surgeon’s activities and intentions to provide the best view rendering camera guidance a non-trivial surgical task. To support the surgeon, a number of robotic systems have been developed to precisely guide the camera in laparoscopic surgery. Some of them, including systems already employed in clinical use [5–9], are telemanipulated by the surgeon allowing full manual control over the field of view at the price of an increased mental workload, thus potentially distracting from critical tasks. To address the limitations of telemanipulated camera guidance, robotic task automation is proposed [12]. Whereas some systems try to sense the intent of the surgeon by monitoring the eye-gaze [13], most of them track the position of the instrument tips and apply basic rules, such as steady instrument following [14]. Limitations of these systems arise due to their lack of flexibility, particularly, as different camera guidance behaviors are required according to various surgical situations. With their limited set of rules, existing robots are unable to adapt to the surgeon’s need, nor do they account for different types of procedures or different surgical steps. However, the attempt to elaborate task specific control schemes requires extensive research even for a limited set of tasks [15].

The limited utilization of autonomous camera guidance during laparoscopic surgery [16] has motivated us to explore the potential of cognitive robotics for surgery. Cognitive robots refer to robotic systems capable of sensing their environment and reacting in a way that is adaptive to the specific situation. Additionally, these robots learn from experience and change their behavior over time [17] with previous applications in service robotics, such as vacuum cleaning [18], or autonomous driving [19]. However, until today this paradigm has not been applied to surgical robotics.

In this work, we demonstrate a novel concept to realize a cognitive model in a surgical robot resulting in cognitive camera control for laparoscopic surgery. Further, we provide proof of robot learning in a surgical scenario leading to the robot improving its performance over time with increased experience. The robotic system is shown to learn not only from human surgical activities, but also from other robots. Additionally, we present an experimental validation of this methodology with two different robot kinematics.

## Materials and methods

### Cognitive model

**Fig. 1:**
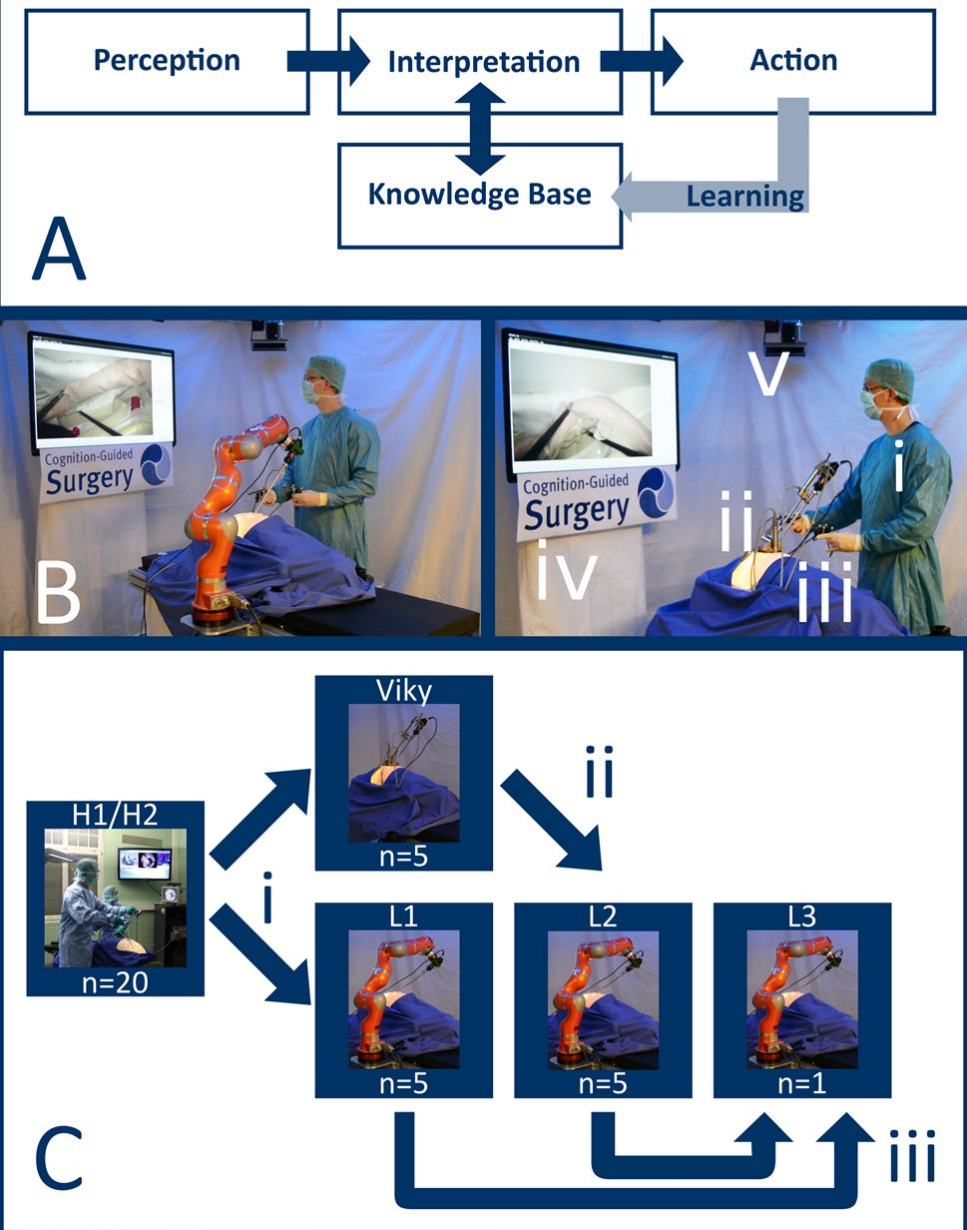
**Learning camera robot.** In the present study the robot realizes a cognitive model (**A**). Here, the robot perceives its environment, interprets it according to a knowledge base and performs a context-aware action. Feedback from that action is incorporated into the knowledge base and enables the robot to learn from experience. For the experimental setup (**B**) either a Light Weight Robot 4 by KUKA Roboter GmbH Augsburg, Germany (LWR, left) or a VIKY EP by TRUMPF Medizin Systeme GmbH+Co. KG, Saalfeld, Germany (right) was used. The surgeon (i) controls the instruments, the robot (ii) controls the minimally invasive camera. Surgery is performed on operation phantom OpenHELP (iii), the endoscopic video is displayed on a screen (iv) while six ceiling mounted cameras track the positions of instruments and minimally invasive camera (vi). The experiments were performed (**C**) with human camera guidance during the surgeon’s learning curve (H1, *n* = 8) and after completion of the surgeon’s learning curve (H2, *n* = 12), Then, learning of the robot occurred during four different experiments in three consecutive steps (adapted from [20]): first, VIKY EP and LWR learned from human demonstration (i). Then, the robot learned from another robot’s experience only (ii). Finally, the robot learned from its own combined experience in two different experiments (iii)

The developed cognitive model combines four elements. The robot *perceived* its environment, *interpreted* it according to a *knowledge base* and performed a context-aware surgical *action*. By incorporating feedback from that action into its knowledge base, the robot was enabled to learn from experience (Fig. 1A). In the following we will give an overview of the system architecture from a surgical perspective. Further technical details can be found in [20].

#### Perception

**Fig. 2:**
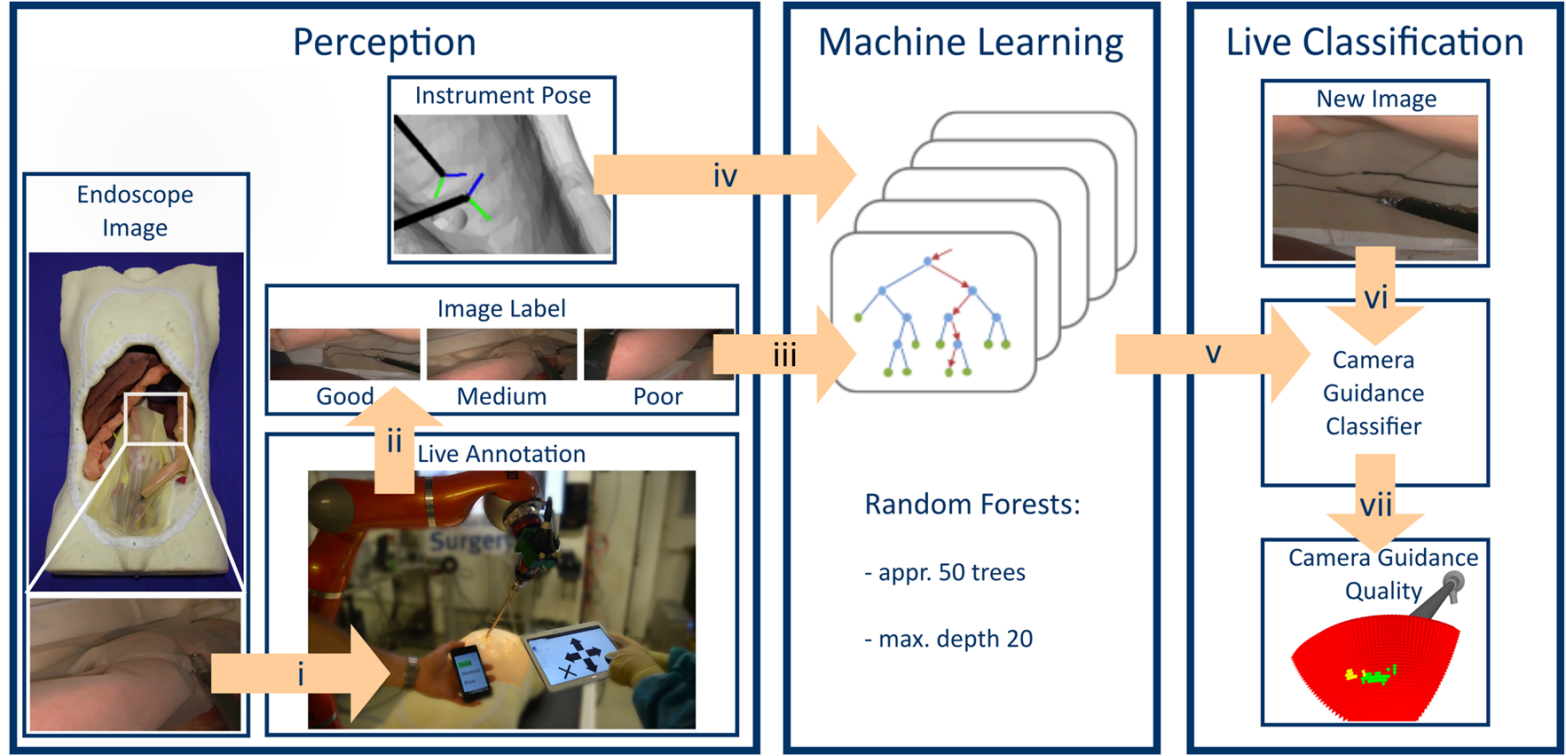
**Classification of camera guidance quality.** For the perception endoscopic image data are collected during the experiments on operation phantom OpenHELP and annotated live with the web interface (i) to generate image labels rating the camera guidance quality “good”, “medium” or “poor” (ii). These image labels (iii) together with the corresponding instrument poses (iv) are used for machine learning. Random forests are used to train camera guidance classifiers (v). The robot applies these classifiers to new images (vi) to calculate camera guidance quality for potential next camera positions for robot motion (vii)

The robot perceived information about its environment from various sensors as well as from a multimodal human–machine-interface (Fig. 2). Utilized sensors include the laparoscopic camera that captures surgical images inside the abdomen, the angle settings in the robot’s joints, as well as external optical tracking to obtain spatial position information of the laparoscopic camera, surgical instruments, and the robot. The human–machine-interface comprised a web interface for mobile devices and the hands-on-robot-mode. The web interface provided means for choosing the surgical step, as well as rating camera guidance quality (see section “Knowledge base”). Additionally, the interface allowed for changing between control modes (cognitive camera control/manual/hands-on) and entering manual movement commands, provided the robot was in manual mode.

#### Knowledge base

The knowledge base constitutes the core element of the cognitive model by replacing the set of movement rules previously used in other autonomous systems, such as visual servoing [21–23]. The knowledge base comprises two types of knowledge, namely factual knowledge and surgical experience (see section “Learning”). For the factual knowledge, three different camera guidance qualities were defined, as summarized in Table 1. Furthermore, the surgical procedure was analyzed similarly to previous work in pancreatic surgery [24]. A surgical process model was created that reflects the experimental setup and comprised of four surgical phases and thirteen surgical steps (Table 2). Additionally, general rules for effective camera guidance were incorporated into the knowledge base, such as the surgical need for a stable horizon.

**Table 1:** Definition of camera guidance quality and evaluation criteria to rate the robot’s performance as feedback for learning

Camera guidance quality	Comfort of surgery	Region of interest	Camera motion
“Good”	Surgeon can perform task comfortably	Central in picture	Not required
“Neutral”	Surgeon can perform task	In picture, not central	Favorable
“Poor”	Surgeon cannot perform task	Not in picture	Required

**Table 2:** Surgical process model for minimally invasive rectal resection with total mesorectal excision in Open Heidelberg Laparoscopy Phantom (OpenHELP)

Surgical phase	Surgical step
Mobilization of colon	Mobilization of sigmoid
	Mobilization of descending colon
	Mobilization of splenic flexure
	Inspection of colon
Dissection of vessels	Lancing retroperitoneum
	Delineating vessels
	Division of artery
	Division of vein
Dissection of rectum	Opening lesser pelvic peritoneum
	Dissection of rectum
Resection of rectum	Transection of rectum
	Salvage of rectum
	Inspection of lesser pelvis

#### Learning

To enable the robot to learn camera guidance from gained experience, and thus, improving its performance, we designed a three-step learning procedure comprising perception of data, annotation of data, and machine learning (Fig. 2). The robot was pre-trained with data obtained from manual camera guidance as performed by a human surgeon (see section “Experimental procedure”). The acquired data for training contained the spatial position of instruments and the camera as well as the camera images. Additionally, a surgical expert retrospectively annotated the data semantically to specify camera guidance quality (Table 1), surgical phase and surgical step (Table 2) for each camera image. Supervised machine learning was then performed on these semantically annotated data to train camera guidance classifiers as models of camera guidance quality [20]. These models were stored as experience within the knowledge base and later used to classify the current camera guidance quality during surgery (see section “Interpretation”). Subsequently, during robotic camera guidance the current surgical step was annotated automatically during robot-assisted cognitive camera control by choosing a camera guidance classifier specific to the respective surgical step. Additionally, the camera guidance quality was rated during the surgery by the surgical expert through the web-interface on a mobile device not interfering with the operating surgeon and the operation itself. New data were used to repeat machine learning to improve camera guidance classifiers and subsequently the robot’s performance.

#### Interpretation

Interpretation included preprocessing of sensor data and classification of camera guidance quality using the camera guidance classifiers in the knowledge base. The instruments’ positions in the camera image were identified via external optical tracking of spatial positions of the laparoscopic camera and surgical instruments. In addition, the endoscope tip position was obtained through the robot’s forward kinematics after hand–eye–calibration as calculated from the joint angle settings. Subsequently, various possible positions of the camera were classified as “good”, “neutral” or “poor” utilizing the previously trained camera guidance classifiers from the knowledge base. Adaptive sampling was performed to make optimal use of the available computing performance. When the current camera position was rated “good”, sampling density was high around this position and only neighboring positions were evaluated. On the contrary, when the camera position was not rated “good”, sampling density was lowered to achieve a higher coverage with the same computational power.

#### Action

The action module actively controlled the robot, i.e. planned the optimal path according to the interpretation of the current situation and executed the planned movements. To smoothen the trajectory and eliminate cyclic camera motions, a time-decaying motion hysteresis was implemented to suppress proposed endoscope positions that were in close proximity to previous ones. Constraining robot motion based on workspace data from human camera guidance ensured avoidance of contact between the camera and the phantom wall. The robot’s control was implemented utilizing the Robot Operating System (ROS) as middleware [25]. During hands-on mode the control software compensated for gravity forces on the robot, however recognizing forces applied by the surgeon’s hand as commands [26]. Furthermore, our implementation allowed operating robots with different kinematics and varying degrees of freedom (DoF) with robots ranging from kinematically deficient (3 DoF) to kinematically redundant (7 DoF).

### Experimental procedure

Figure 1B gives an overview of the experimental setup.

#### Phantom model

Minimally invasive rectal resection with total mesorectal excision was chosen to experimentally evaluate cognitive camera guidance, as this surgical procedure places high demands on camera control. Surgery takes place in three quadrants of the abdomen and in the lesser pelvis requiring different types of camera guidance. For example, the “mobilization of sigmoid” requires steady, but long movements in one direction, whereas the “dissection of rectum” requires a rather dynamic camera guidance following the instruments in the narrow space of the lesser pelvis. The procedure was executed on a previously introduced standardized, human-sized model for minimally invasive surgery (Open Heidelberg Laparoscopy Phantom, OpenHELP) [27]. In our experiments the original torso, liver, spleen, kidney, bladder, pelvic floor muscles, and rectum were modeled and included in the OpenHELP. Additionally, a colon was created from cloth, filled with cotton wool and covered with latex sheets simulating visceral peritoneum and mesocolon. Furthermore, aorta, inferior mesenteric artery and vein with connective tissue, ureter and peritoneum covering these structures were added. Several of these structures, including mesocolon, peritoneum, inferior mesenteric artery and vein, were cut during experimental surgery, and thus, were rendered replaceable. The 3D printed abdominal wall mimicked the pneumoperitoneum and laparoscopy was performed through a set of holes representing standard trocars.

#### Medical devices and instrument tracking

For experimentation, we utilized one of two minimally invasive cameras with 0° optics (TIPCAM1 3D with IMAGE1 camera control, KARL STORZ GmbH & Co. KG, Tuttlingen, Germany and R. Wolf Endocam Logic HD, Richard Wolf GmbH, Knittlingen, Germany). Surgical instruments included a minimally invasive bowel grasper, bowel grasper with gauze, scissors, and single clip applier (KARL STORZ GmbH & Co. KG, Tuttlingen, Germany). Images from the minimally invasive camera were recorded with an average frame rate of 27 Hz on a personal computer (Intel Core i5-4590, 32GB RAM, SSD HDs, Blackmagic DeckLink Mini Recorder) connected to the respective camera control unit. Optical tracking devices tracked the positions of surgical instruments and robot through external, passive optical tracking. We either applied two Polaris® systems (Northern Digital Inc., Ontario, Canada) with one dynamic reference frame for registration of both coordinate frames, or an ARTTRACK system (Advanced Realtime Tracking GmbH, Weilheim i.OB, Germany) with six ceiling-mounted cameras.

#### Robot kinematics

During experiments, generalizability of our cognitive model was conveyed through utilizing two different robot kinematics. The VIKY EP (TRUMPF Medizin Systeme GmbH+Co. KG, Saalfeld, Germany) was used with a modified control unit to enable remote control of the robot. This 3 DoF robot with no additional sensors is certified for medical usage. The second utilized robot was the LWR 4 (KUKA Roboter GmbH, Augsburg, Germany) with 7 DoF and additional kinematic sensors for safety and hands-on mode, however not certified for medical usage. During experimental evaluation, both robots were attached to the operation table by metal brackets opposite to the surgeon, thus preventing collision with the surgeon.

#### Experimental evaluation

A number of different experiments consisting of several operations each were performed to train the robot based on human camera guidance, allow it to learn from experience, and investigate improvements in the robot’s performance with additional experience. All surgeries were performed by the same surgeon who had completed his learning curve with the operative procedure in the phantom model prior to robot usage. As illustrated in Fig. 1C, a total of *n* = 20 operations were performed with human camera guidance by the same camera assistant. Experiments H1 (*n* = 8) and H2 (*n* = 12) refer to procedures prior to and after completing the learning curve, respectively. Human camera guidance was followed by experiment Viky (*n* = 5) and experiment L1 (*n* = 5) investigating cognitive camera control and using the knowledge base of H1 and H2. Subsequently, experiment L2 (*n* = 5) was conducted with LWR, however, only utilizing data generated with VIKY EP (experiment Viky) for learning camera guidance classifiers. Hereby, we demonstrated cross-robot learning, i.e. the robot’s ability to learn from another robot’s experience with varying kinematics instead of human training. Finally, for experiment L3 (*n* = 1), data from all previous procedures with LWR (experiments L1 and L2) were used for learning to demonstrate continuous learning.

An approval by our institutional review board and written informed consent were not necessary, as there was no study on human or animal subjects performed.

### Statistical analysis

We evaluated the robot’s performance during each operation through the objective parameters “duration of the surgery”, “proportion of cognitive camera control as opposed to manual or hands-on mode”, as well as “required amount of alternating between cognitive camera control to manual or hands-on mode”. As a subjective parameter “camera guidance quality” was used by calculating the proportion of good/neutral/poor. For all parameters, mean and standard deviation were calculated. To investigate robot learning between experiments L1, L2 and L3, Levine’s test for homogeneity was performed, followed by a one-way analysis of variance (ANOVA). To test our hypothesis of a learning robot, contrasts for L2 versus L1 and L3 versus L2 were provided with a *p* value < 0.05 considered to prove statistical significance. Data analysis and visualization were performed with R statistics [28].

## Results

The cognitive camera control was realized successfully as demonstrated in movie S1. In total *n* = 36 operations over a duration of 977 mins, 807’816 frames and 1’845’607 spatial positions of camera and instruments were recorded. Additionally, 22’628’255 synthetic 3D positions of the camera were generated as training samples.

**Fig. 3:**
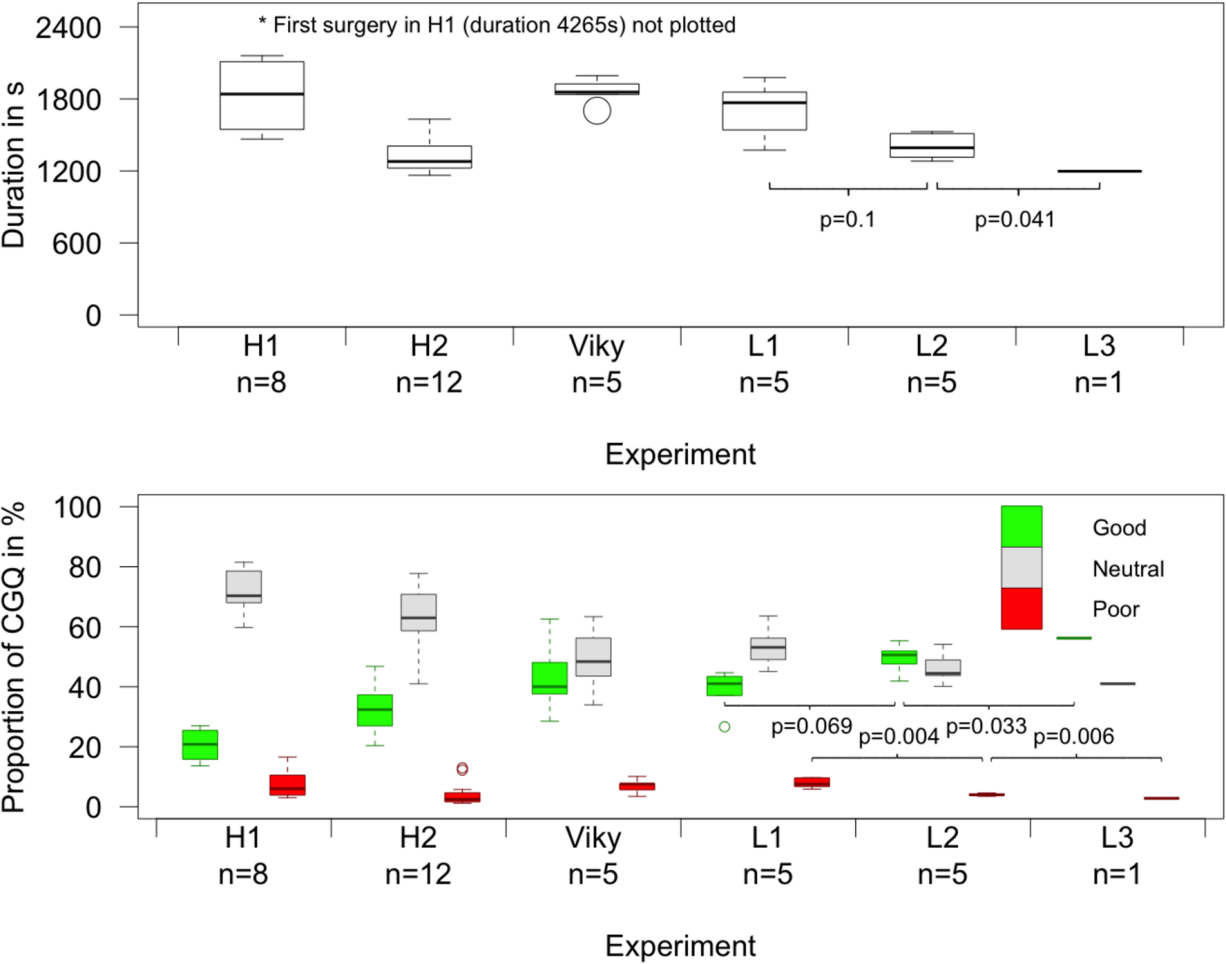
**Experimental results.** Human camera guidance (*n* = 20) is divided to account for the initial learning curve of the surgeon with the phantom setup (H1, operations 1–8) and human camera guidance after completion of the learning curve (H2, operations 9–20). The other groups represent robotic camera guidance with VIKY EP learned from human (Viky), Light Weight Robot 4 (LWR) learned from human (L1), LWR learned from Viky (L2) and LWR learned from L1 and L2 (L3). The upper boxplot displays the duration of the experiments (adapted from [20]), the lower boxplot displays the camera guidance quality (CGQ) of the experiments, i.e. the proportion of camera guidance being rated good/neutral/poor by a surgical expert. *p* values have been determined by one-way analysis of variance

The duration of the operation was influenced by an initial learning curve of the operating surgeon during human camera guidance (H1, operations 1–8). The learning curve was determined based on a distribution of durations with a maximum of 4265 s (operation 1) decreasing to 1465 s (operation 8) and an average duration of 2090 ± 919 s. After completion of this learning curve, the average duration of one operation procedure was 1325 s ± 144 s for human guidance (H2, operations 9–20). The duration of the operation supported by cognitive camera control decreased with increasing experience of the robot from L1 (1704 s ± 244 s) over L2 (1406 s ± 112 s) to L3 (1197 s), indicating a learning curve for the robot. Here, Levine’s test shows homogeneity of variances for experiments L1, L2 and L3. One-way ANOVA resulted in a significant difference between the individual experiments (*F*-value = 4.7, *p* = 0.045). However, the comparison between operation duration for L1 and L2 did not reach statistical significance (*p* = 0.1). The resulting durations for human and robotic camera guidance are summarized in Fig. 3A.

Camera guidance quality rated as “good”, “neutral”, “poor” by a surgical expert improved with increasing experience of the robot, such that a higher proportion of “good” and lower proportion of “poor” was observed from L1 over L2 to L3. Levine’s test showed homogeneity of variances for L1, L2 and L3 for both, “good” and “poor” camera guidance. One-way ANOVA showed a significant difference between the experiments (“good”: *F*-value = 5.52, *p* = 0.031; “poor”: *F*-value = 14.58, *p* = 0.002). However, for the proportion of “good” camera guidance the difference between experiments L1 and L2 did not reach statistical significance. The resulting camera guidance quality for human and robotic camera guidance is summarized in Fig. 3B.

For the proportion of cognitive camera control compared to manual or hands-on mode Levine’s test showed homogeneity of variances for L1, L2 and L3. One-way ANOVA showed a significant difference between the experiments (*F*-value = 34.2, *p* < 0.001). The proportion of cognitive camera control increased with increasing experience from L1 (62.4% ± 4.9%) to L2 (85.4% ± 4.2%, *p* < 0.001) and L3 (85.1%, *p* = 0.002). Accordingly, the number of times the surgeon had to change to manual or hands-on mode to allow for manual commands to the robot during surgery decreased from L1 (21.8 ± 3.7) to L2 (10.4 ± 2.1, *p* < 0.001), but not for L3 (14, *p* = 0.45). Levine’s test showed homogeneity of variances for L1, L2 and L3. One-way ANOVA showed a significant difference between L1, L2 and L3 (*F*-value = 18.3, *p* = 0.001).

## Discussion

Our primary achievement is the demonstration of a methodology that represents a paradigm shift from previously programmed robots not suitable to adapt to a surgeon’s needs to a new generation of cognitive robots that will be able to adapt to different surgeons, surgical situations and surgical procedures. Our methodology enables cognitive camera control for a learning robot in minimally invasive surgery. The methodology is applicable to various robot kinematics as demonstrated during experimental validation. Our experiments showed the robot’s ability to learn from human surgeons, from other robots, and on its own.

As early as 2008 the SAGES-MIRA Robotic Surgery Consensus Group envisioned that “Robots could use artificial intelligence to learn from the surgeon operating the device” [29]. In our study the robot positioned itself autonomously after having learned the desired spatial relation of surgical instruments and camera, depending on the phase of a surgical procedure. This autonomy of the robot to position the camera enabled the surgeon to focus on essential surgical tasks, without the need to guide the camera. If the camera position was unsatisfactory to the surgeon, he was able to correct it manually using one of different input methods (touch display or hands-on mode on the LWR). In contrast to previous systems, the robot extended its knowledge base and learned from this experience for future interventions. Thus, the robot changed its behavior over time and improved its performance with every additional procedure resulting in adaptation of the robot to the surgeon and not vice versa. Thereby, different behavioral patterns and individual preferences of various surgeons may be learned by the robot, presenting a matter for future investigation. In addition, due to the provided surgical process model, it was possible to train camera guidance classifiers tailored for each surgical step. The classifiers may also be tailored to the individual surgeon’s preferences over the course of several interventions. Based on the duration of procedures with human camera guidance, we can assume that the learning curve for the surgeon had been completed before initiating the robotic experiments. Thus, a difference in the overall performance of the team “surgeon plus robot” was likely to be caused by the improved robot performance. Here, we focused on the robot learning curve as the difference between the three experiments L1, L2 and L3. The resulting learning curve was characterized by decreasing duration of the surgery with higher proportion of “good” camera guidance quality and lower proportion of “poor” camera guidance quality. Furthermore, less manual corrective feedback was required for L2 and L3 compared to L1. Moreover, apart from the quantitative results the robot proved learning in a qualitative way as perceived by the surgeon. Initially, during several surgical steps, such as opening the lesser pelvic peritoneum, the zooming motion (i.e., moving the camera in or out) was “trained” explicitly, because during experiments Viky and L1 no zooming was conducted by the cognitive camera control. Instead, the robot was manually directed to zoom in when required by the surgeon. Subsequently, in experiments L2 and L3, the robot zoomed in autonomously as it had learned the necessity for this movement from data collected during Viky and L1.

Metrics to measure the quality of the camera guidance and the robot’s learning progress limit the evaluation of learning camera robots. Moreover, only few of these metrics can be utilized as a learning feedback for the robot. Still, feedback to the robot is of utmost importance as better feedback results in better surgical training of the robot [30, 31]. The objective metrics overall procedure time and setup time are a common standard for evaluation of surgical robots. These metrics can be calculated automatically by the robot’s software and are easily comparable (at least for simple surgical procedures). Their main disadvantage is the lack of specificity in the feedback for a learning robot. Thus, in our approach, duration of the surgical procedure was not implemented as a feedback for the robot, but only to measure and evaluate its performance. However, reduced procedure time may also result from the surgeon learning to work with the robot. Subjective metrics, such as questionnaires focusing on the surgeon’s user experience are crucial for user acceptance, and thus, the translation into practice. Unfortunately, most criteria are not well defined and the results of these criteria are not entirely representative for all surgeons as they strongly depend on personal preferences. Additionally, these criteria have to be obtained after the surgical intervention increasing the workload of the surgeon and feedback often is not detailed enough to improve the system’s performance in specific situations. To overcome some of these limitations, we used camera guidance quality as an additional metric. During experimentation, an additional surgical expert assessed the camera guidance quality, rendering it a direct subjective metric. Nevertheless, this metric has proven to be suitable as feedback to a learning robot. The disadvantage of rating the camera quality during experiments is the necessity of a surgical expert in addition to the surgeon, because annotation by the surgeon would interfere with the surgical workflow. Thus, the number of interactions of the surgeon with the system was logged and evaluated, indicating the surgeon’s satisfaction with the (autonomous) camera guidance with less interaction (or correction) representing better outcomes. In future investigations we would like to use these corrective commands directly as a feedback for the learning robot. This would also diminish the time-consuming annotation of camera guidance quality be an additional surgical expert.

Main limitation of our study is its limited number of participants. For this feasibility study a single surgeon performed all operations rendering the procedures standardized and easy to compare, especially for investigating the robot’s learning curve, at the expense of generalization of the results. Additionally, the robot’s capabilities were only evaluated during one surgical procedure. Even though laparoscopic rectal resection is a rather complex procedure, it has yet to be proven, whether the approach is applicable to other procedures (cholecystectomy, hernia repair, pancreatic surgery etc.). Finally, our study is limited to a medical phantom setuo awaiting translation into animal lab studies and subsequent clinical trials. Furthermore, although most surgeons prefer a 30°-optic for laparoscopic anterior rectal resection with total mesorectal excision, in our study we utilized a 0°-optic. Usage of a 30°-optic would result in an additional degree of freedom in camera control posing another major challenge for the machine learning algorithm. Thus, further engineering and computer science research are necessary to fulfill this surgical requirement in future work. In addition, our approach has to proof not only feasibility, but also advantages over previous approaches of visual servoing, i.e. simply following the instrument tip with the camera. Nevertheless, the present study not only demonstrates a completely novel cognitive approach to camera guidance robots and their application in a surgical setting, but also demonstrates the robot’s learning progress and improvement in performance.

Cognitive surgical robots may have a huge clinical impact in the future because of their ability to perceive their environment, interpret the situation based on a knowledge base, and act accordingly. These robots can learn and improve their performance by incorporating new experiences into their knowledge base adapting to different surgeons, different surgical procedures and different patient anatomies. Future research may focus on incorporation of existing or novel technologies, such as surgical phase detection and autonomous manipulation of tissue. In the present study, external cameras continuously track the instruments, however, it has previously been shown that instruments as well as organs can be tracked directly in the laparoscopic video by means of computer vision [32]. Whereas we defined the phase of the operation manually during the operation, research suggests that skilled surgeons can extract it from surgical device data alone [33] leaving room for further automated detection of the surgical phase. For simple procedures, such as cholecystectomy, the phase can even be automatically recovered from the video using deep learning algorithms [34] and procedure duration can be recovered from medical device data in real time [35]. Furthermore, reinforcement learning algorithms are a current trend to solve complex motion tasks in robotics. However, employing reinforcement learning algorithms, that require interaction between learning robot and environment, poses a severe safety issue in medical robotics. Clinical translation of the proposed learning camera guidance system may lay the foundation to gather high quality and quantity of labeled data to employ offline reinforcement learning algorithms to the task of robotic camera guidance to gradually learn and represent more complex behaviors [36]. In a recent review of machine learning techniques in surgical robotics Kassahun et al. describe a number of applications for learning surgical robots, such as tying a knot or steering of vascular catheters [37]. They conclude that machine learning may help to extract human skill and transfer it to surgical robots emphasizing the potential of cognitive surgical robots as demonstrated in our study. Furthermore, the concept of a robot learning from another robot’s experience that we demonstrated in our study, may help to transfer experience easily from one surgical center to another via data transfer instead of having to train the robot all over again.

In the future, cognitive surgical robots and automated systems may become the center of a fully integrated cognitive operating room. They will not only guide the camera or steer catheters, but may also expose tissue with the right force, cut in the correct surgical plane [38], and reconstruct anastomoses after resection [39]. Perception may not only be based on visual information in the laparoscopic image, but also on multimodal data from medical devices and vital parameters from patient monitoring. If the combination of these data with powerful machine learning algorithms continues as outlined in the concept of Surgical Data Science [40], within 15 years cognitive surgical robots will not only enhance human surgeons’ capabilities. They will likely perform autonomous tasks and may even perform simple surgical procedures autonomously, thus changing the face of surgery.

## Supplementary Information

Below is the link to the electronic supplementary material.

Supplementary file1 (MP4 56102 kb) **Movie S1.** Demonstration of robot-assisted cognitive camera control in minimally invasive surgery.
